# Supplementary material for: Digital finance and M&As: An empirical study and mechanism analysis
Source: PLoS One. 2023 Aug 10;18(8):e0289845. doi: 10.1371/journal.pone.0289845 (PMC10414609; doi:10.1371/journal.pone.0289845)
Supplement: Attachment — Submitted filename: Response to reviewers R2.docx [file pone.0289845.s003.docx]

**Rebuttal letter**

**Dear editor:**

Thank you so much for your advice on the manuscript entitled “**Digital finance and M&As: An empirical study and mechanism analysis**” (Manuscript ID: PONE-D-23-09479). Your comments are very valuable and helpful for revising the paper and improving the quality. As you mentioned, this paper referred retracted articles. We have substituted these two references with other articles, please have a check. Let me know if there are further questions.

**Dear reviewer 1:**

Thank you so much for your comments about the abstract, which is quite beneficial for improving the paper quality. We are happy to know to that our revised paper meets the criteria.

**Dear reviewer 3:**

Thank you so much for your comments on the manuscript entitled “**Digital finance and M&As: An empirical study and mechanism analysis**” (Manuscript ID: PONE-D-23-09479). Your comments are very valuable and helpful for revising the paper and improving the quality. Details of revision are provided below.

1. Revise the paper again to reduce the grammar and punctuation imperfections;

Response: Thanks for the comments, we have revised the paper thoroughly again, please have a look.

2. It would be beneficial to extend the discussion section and compare your outputs and findings in a broader context.

Response: Thanks for the comments, the discussion has been extended as followed:

**5. Further discussion**

Considering the heterogeneity of digital financial structure, the imbalance of regional economic development, and differences in enterprises’ equity nature and industries, these may lead to great differences in the impact of digital finance on M&As activities in different enterprises. Therefore, this research divides digital finance into three dimensions and classifies the samples into several categories according to regions, equity nature, and sector differences to do further analysis.

5.1 The heterogeneity of digital financial structure

The digital finance index covers three sub-dimensions including coverage breadth, usage depth, and digitalization level. Coverage breadth reflects the regional financial environment by counting the number of people using electronic accounts. Usage depth describes the regional financial services’ ability through the usage of various digital financial services while digitalization level measures the cost and efficiency of digital finance. In order to more accurately examine which dimension plays a more significant role on enterprises’ M&As activities, we carry out structural analysis of digital finance. The results are shown in Table 7.

**Table 7 Results of the heterogeneity of digital financial structure**

| Variable | Model (10) | Model (11) | Model (12) |
| --- | --- | --- | --- |
| coverage | 0.0223***  (60.84) |  |  |
| usage |  | 0.0116***  (46.34) |  |
| digitalization |  |  | 0.0171***  (57.80) |
| Industry | Yes | Yes | Yes |
| Time | Yes | yes | Yes |
| Control | Yes | Yes | Yes |
| Constant | -6.3738***  (-16.20) | -6.8040***  (-18.03) | -5.3799***  (-13.74) |
| Obs. | 10883 | 10883 | 10883 |
| R^2^ | 0.1969 | 0.1102 | 0.1766 |

Note: (1) ***, **, and * represent significant at 1%, 5%, and 10% levels respectively. (2) The values in parentheses are Z values.

It can be found that in Table 7, the significances of all these three dimensions reach the 1% significant level. Further, we conduct the seemingly unrelated estimation test (SUEST) to compare whether differences in coefficients exist. As SUEST can only compare whether the coefficients differed between two groups [41], we perform pairwise comparisons between the coefficients of coverage, usage, and digitalization. Results in Table 8 indicate that the p values are all significant at 1% level while the coefficients of these three dimensions are 0.0223, 0.0116, and 0.0171 respectively. Therefore, it can be concluded that coverage breadth plays a much stronger role, followed by digitalization level. The breadth of digital financial coverage is more conducive to improving broaden the boundaries of financial services and from the perspective of efficiency, coverage breadth is the most direct way to benefit enterprises in remote areas. Therefore, more digital infrastructure should be constructed at the early stage, through expanding the coverage of digital services to strengthen usage depth and promote digitalization level. [42] also proved that the larger the coverage breadth is, the more customers can be covered, which is also proved by [43].

**Table 8 Results of SUEST test**

| Variable | Usage | Digitalization | East | SOE | Second | Third |
| --- | --- | --- | --- | --- | --- | --- |
| Coverage | 0.000*** | 0.000*** |  |  |  |  |
| Usage |  | 0.000*** |  |  |  |  |
| Non-east |  |  | 0.000*** |  |  |  |
| Private |  |  |  | 0.001*** |  |  |
| First |  |  |  |  | 0.1193 | 0.2220 |
| Second |  |  |  |  |  | 0.1287 |

Note: (1) ***, **, and * represent significant at 1%, 5%, and 10% levels respectively.

5.2 Region, digital finance and M&As

The unbalanced distribution of financial resources broadens the economic gap between different regions, which leads to the east more developed while the central and west being less developing. Besides, according to “China regional financial operation report (2021)” [44] issued by The People's Bank of China, the number of banking financial institutions in western and middle China was 59,000 and 53,000 respectively, compared with more than 100,000 in the east. As a result, the financial demand of most enterprises especially in west and central areas cannot be met.

According to the criteria for the division of east, west, and central areas in China issued by the National Bureau of Statistics, we divide samples into two categories based on the regions where companies registered. 0 is given to companies located in eastern region, while central and western areas are set as 1. Based on the analysis above, this paper proposes Hypothesis 3: The influences of digital finance on M&As activities are more significant for enterprises located in west and central areas.

As Column (1) - (2) shown in Table 9, it can be found that the significances of both east and non-east areas are at 1% significant level. SUEST test indicates that the p value is 0.000 and significant at 1% level, which proves the influences various on different areas. Since the coefficients of east and Midwest areas are 0.0379 and 0.1008 separately, it can be concluded that the effects of the development of digital finance in promoting M&As activities are much stronger in the west and central area. Thus, H3 is approved. After receiving the same level of financial supports, Midwest enterprises are more likely to implement M&As activities. Therefore, the promotion of the development of digital finance is important for narrowing the regional financial gap and give Midwest companies more opportunities for achieving sustainable development and industrial transformation. The findings are also consistent with [20], [45-49].

**Table 9 Regional heterogeneity test results**

| Variable | Region | | Equity nature | | Sector | | |
| --- | --- | --- | --- | --- | --- | --- | --- |
|  | (1)  East | (2)  Non-east | (3)  SOE | (4)  Private | (5)  First | (6)  Second | (7)  Third |
| DF | 0.0379***  (62.23) | 0.1008***  (38.21) | 0.0253***  (34.05) | 0.0287***  (55.87) | 0.0431***  (6.38) | 0.0268***  (52.99) | 0.0281***  (37.53) |
| Control | Yes | Yes | Yes | Yes | Yes | Yes | Yes |
| Industry | Yes | Yes | Yes | Yes | Yes | Yes | Yes |
| Time | Yes | Yes | Yes | Yes | Yes | Yes | Yes |
| Constant | -8.3418***  (-15.36) | -9.7294***  (-6.50) | -4.7314***  (-6.07) | -7.3064***  (-12.85) | -1.7503  (-0.29) | -9.3662***  (-8.60) | -5.1855***  (-5.76) |
| Obs. | 8098 | 2785 | 3217 | 7666 | 101 | 7101 | 3681 |
| R^2^ | 0.3105 | 0.5964 | 0.2120 | 0.2494 | 0.3032 | 0.2322 | 0.2436 |

Note: (1) ***, **, and * represent significant at 1%, 5%, and 10% levels respectively. (2) The values in parentheses are Z values.

5.3 Equity nature, digital finance, and M&As

[50] suggest that the financial system in China is dominated by the banking system which is controlled by the government indeed. Although state-owned enterprises may suffer from financing problems to some extent, due to financial subsidies and policy supports they received from the governments, state-owned enterprises are much easier to get financing. Besides, private companies are mainly small and medium-sized compared with state-owned enterprises, which means they have fewer assets and collateral. Furthermore, private enterprises have lower management efficiency and quality of financial information. Thus, it is difficult for private enterprises to get funds from traditional financial markets. While the development of digital finance can attract more investors and provide more targeted financial products and services at the same time, the financial demands of private enterprises can be met to a larger extent. Therefore, this paper believes private enterprises are more likely to conduct M&As activities if financial constraints can be alleviated. Similarly, the value of 0 is given to state-owned enterprises, otherwise, the variable is set as 1. Accordingly, we propose Hypothesis 4: Compared with state-owned enterprises, the positive effects of the development of digital finance on M&As activities are more prominent for private enterprises.

Column (3) and (4) in Table 9 presents the results of the impact of digital finance on M&As activities across companies with different equity nature. It shows that the significance of both state-owned and private enterprises is at the 1% level and the p value of SUEST test is 0.001, which is positive and significant at 1% level. The coefficients of state-owned and private enterprises are 0.0253 and 0.0287 individually, therefore, the development of digital finance put greater influence on private enterprises as their financial demands are much larger, thus supporting H4. Private enterprises make up an important role of China’s economy and the digital finance enhance their access to financial services. As a result, private enterprises operate and produce more efficiently, which leads to a more dynamic economy. The result is also consistent with [51-54].

5.4 Sector, digital finance, and M&As

According to “Guidelines for the classification of listed companies in China” (2012 Version) [55], we divide samples into three categories based on industry attributes and do regression tests. Specifically, companies in category A are classified into primary sector mainly agricultural enterprises in China. Industrial manufacturing industry enterprises belonging to category B are considered as secondary sector. The remaining enterprises are regarded as third sector. Currently, agricultural enterprises are at the mature stage and have lower capital demands, providing funds for banks and other financial institutions instead. On the contrary, industrial companies need plenty of funds to operation to achieve sustainable development and industrial transformation. Also, most of the non-financial tertiary industries are private enterprises and companies’ sizes are relatively small, which makes them difficult to get funds from traditional financial institutions. Besides, primary industry has fewer M&As activities compared with secondary and third industries due to their operation strategies. Thus, we propose Hypothesis 5: The positive effects of the development of digital finance on M&As activities are less prominent on primary industry compared with secondary and third industry.

The regression results are presented in Column (5), (6), and (7) of Table 9. It can be found that the significances of primary, secondary, and tertiary industry are all at 1% level with the coefficients of 0.0431, 0.0268, and 0.0281 respectively. However, SUEST test in Table 8 shows that none of the p values are significant with the figures of 0.1193, 0.2220, and 0.1287 individually, which means there is no difference in the impact of digital finance on different sectors. Thus, Hypothesis 5 should be not accepted. One possible explanation is that, according to statistics, the number of M&As in the primary industry is relatively small compared with the other two industries. After setting several sample selection criterions, the eligible sample size is only 101, which is not large enough to find the difference across sectors and may lead to variations in results. While a recent research [56] indicates that the impact of the development of digital finance on M&As activities varies across industries. To be more specific, the positive effects of the development of digital finance on M&As activities are more prominent in the tertiary industry with the primary coming to the second. However, their findings still differ from our hypothesis. Thus, it points out the way for future analysis.
